# Assessing the Benefits of a Novel Postural Supporting Device on the Postural Development of Preterm Neonates in the Neonatal Intensive Care Unit: A Feasibility Study

**DOI:** 10.7759/cureus.86052

**Published:** 2025-06-15

**Authors:** Dhwani D Chanpura, Neha Mukkamala, Nalina Gupta

**Affiliations:** 1 Physiotherapy, Sumandeep Vidyapeeth Deemed to be University, Vadodara, IND; 2 Neurological Physiotherapy and Community Rehabilitation, Sumandeep Vidyapeeth Deemed to be University, Vadodara, IND

**Keywords:** nicu, position device, positioning, postural development, preterm

## Abstract

Advances in neonatal intensive care have significantly increased the survival rates of preterm infants. However, many of these infants continue to face serious motor impairments, contributing to both short- and long-term morbidity. Therapeutic positioning is a key neurodevelopmental intervention in neonatal intensive care units (NICUs), aimed at supporting posture and movement. This feasibility study evaluated the effects of a novel postural support device on the postural development of preterm neonates in the NICU. Nine medically stable preterm infants, with no diagnosed diseases or conditions, were included in the study. All participants received daily neonatal physiotherapy and were continuously positioned in the novel postural support device for one week. The Infant Positioning Assessment Tool was used to assess posture before and after the intervention. After one week, a statistically significant improvement was observed across all posture components - head, neck, shoulders, hands, hips/pelvis, and knees/ankles/feet - with a p-value of 0.026. These findings suggest that the novel postural support device, in combination with physiotherapy, positively influences the postural development of preterm neonates.

## Introduction

Preterm neonates, defined as infants born before 37 weeks of gestation, face significant developmental challenges due to the immaturity of their neurological and musculoskeletal systems [1]. While advances in medical technology have greatly improved the survival rates of preterm infants, this has also led to increased rates of both short- and long-term morbidity, often stemming from brain malformations or injuries during critical periods of development [2]. Approximately 5-10% of preterm neonates are at risk for complications such as intraventricular hemorrhage, periventricular leukomalacia, and cerebral palsy - conditions frequently associated with visual, auditory, intellectual, behavioral, and sensory processing impairments [3].

The initial and most significant challenge for preterm neonates is adapting to life outside the womb, a process made more difficult by their underdeveloped nervous systems [4]. In the final trimester of pregnancy, the amniotic fluid provides a cushioned environment that promotes a naturally flexed posture, with the arms and legs tucked toward the center of the body. After birth, this supportive intrauterine environment is replaced by open space with minimal physical containment, making it harder for preterm infants to maintain a flexed position. As a result, they are more prone to abnormal postures, which disrupt neuromuscular development and contribute to muscle imbalances, poor joint alignment, and delayed motor milestones [5]. Motor impairments are notably more prevalent in preterm than full-term infants, affecting only 2-7% of full-term neonates compared to 54-64% of those born preterm. These impairments manifest in spontaneous movements that are less fluent, less varied, and poorly coordinated [6].

Stable posture is considered critical for effective motor planning and coordination [4]. However, preterm neonates are particularly sensitive to gravity, which can negatively affect their posture and alignment. Lacking the supportive boundaries of the womb, they often adopt hyperextended positions due to low muscle tone. In this posture, the trunk and limbs extend against the flat surfaces they lie on, leading to asymmetrical body positioning and movement patterns [6,7].

Promoting physiological flexion through therapeutic positioning is a key neurodevelopmental strategy in neonatal intensive care unit (NICU) care. This approach supports optimal joint and skeletal alignment, enhances muscle tone, improves feeding abilities, and fosters self-regulation and comfort [8]. Various positioning methods, such as swaddling, nesting cushions, positioning rolls, and specialized devices like the Snuggle Up, Bendy Bumper, and Dandle Roo, are commonly used in the NICU [9]. However, traditional positioning aids often lack the firmness needed to properly support the infant, sometimes allowing the legs to fall outward, which can impede motor development. Conversely, while advanced devices offer better structural support, they tend to be expensive and less accessible in low-resource settings [8,9].

Given the routine use of positioning devices in NICU care, there is a clear need for a new postural support device that addresses the limitations of current options. This study was conducted to examine the effects of a newly developed postural support device on the postural development of preterm infants. The Infant Positioning Assessment Tool (IPAT), a standardized evaluation instrument, was employed to assess and monitor postural changes over time.

## Materials and methods

This feasibility study was approved by the institutional ethics committee (SVIEC/ON/PHYS/PhD/22012) and conducted in the NICU of Dhiraj General Hospital, Vadodara, India, from June to August 2024. Written informed consent was obtained from the caregivers or guardians of all enrolled preterm neonates prior to the commencement of the study.

Preterm neonates were included based on specific criteria: gestational age between 30 weeks 0/7 days and 36 weeks 6/7 days, medical stability within the first 72 hours of life, and referral for physiotherapy. Exclusion criteria included the need for oxygen therapy or any form of respiratory support, such as mechanical ventilation, continuous positive airway pressure, or high-flow nasal cannula, beyond 72 hours after birth, as well as any diagnosed neurological, musculoskeletal, genetic, chromosomal, or metabolic disorders.

The aim of this feasibility study was to explore the preliminary effects of a newly developed postural support device on the postural development of preterm neonates. The primary objectives were to assess the practical implementation, acceptability, safety, and early impact of the intervention, rather than to test a hypothesis or identify statistically significant group differences. Based on recommendations in the literature, a sample size of six to 12 participants is considered appropriate for feasibility and pilot studies [10,11]. Accordingly, nine preterm neonates were enrolled. The recruitment rate was 75% of eligible neonates during the study period, and retention was 100%, with no dropouts or withdrawals.

Before initiating the intervention, each neonate underwent a detailed physiotherapy assessment to establish baseline data. Postural quality was assessed using the IPAT, a validated clinical instrument originally developed by Coughlin et al. [12]. The IPAT was used in its standard form, without any modifications, to evaluate outcomes in both the control and intervention groups. IPAT scores were recorded and analyzed to determine the intervention’s effect on postural development.

All neonates received daily neonatal physiotherapy sessions lasting approximately 20 minutes and were continuously positioned in the novel postural support device for 24 hours a day over one week. This intervention followed recommendations by the American Physical Therapy Association [13,14]. Parents were included in treatment sessions and were encouraged to perform the recommended activities during their NICU visits. The intervention was carried out during the infant’s wakeful state, ideally before the next scheduled feeding. It was paused or stopped if the neonate showed signs of stress, such as fussiness, crying, or sleep. Throughout the intervention, neonates were closely monitored to promptly address minor concerns, including skin integrity issues, desaturation episodes, or signs of discomfort. These concerns were managed immediately by adjusting or halting the intervention, ensuring continuous safety and comfort. None of the neonates exhibited any adverse signs.

As part of the study, a novel postural support device was designed by the authors specifically for preterm neonates in the NICU. The goal was to replicate the natural flexed intrauterine posture. The device was constructed using medical-grade memory foam and soft cotton and included adjustable components to help maintain optimal positioning. It supported the head and neck in the midline, stabilized the shoulders, and maintained the hips and knees in a flexed, developmentally appropriate position. The device measured approximately 25 inches in height and 19 inches in width, dimensions based on ideal anthropometric data published by the Indian Academy of Pediatrics. All components, including head and neck support, shoulder rolls, and knee bolsters, were interconnected using Velcro, allowing for easy adjustment to fit smaller infants. During use, the components were arranged on the device base and covered with a sterile hospital green drape to ensure hygiene and comfort.

**Figure 1:**
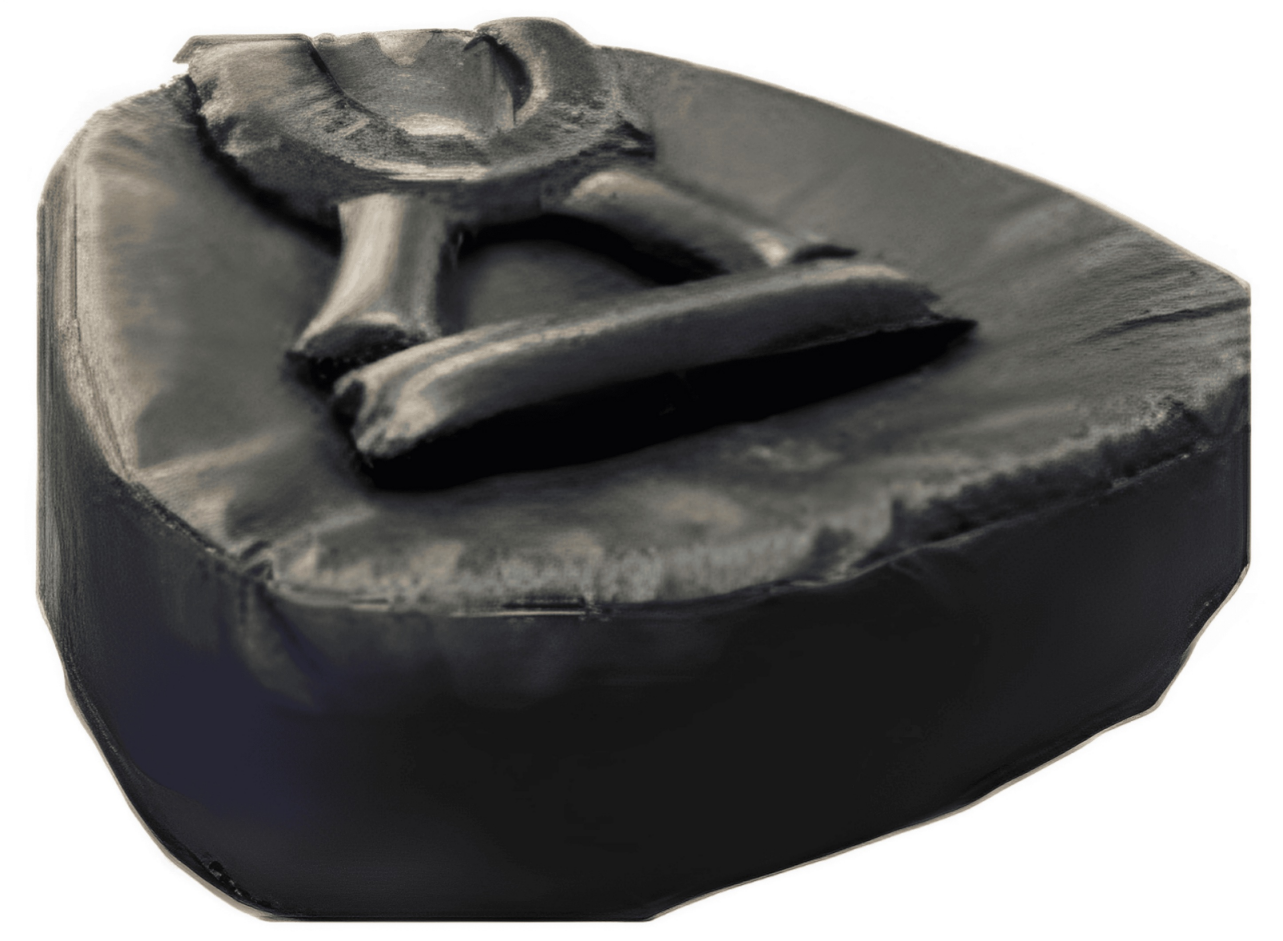
Postural supporting device Image credits: Dhwani D. Chanpura and Nalina Gupta

The device underwent thorough testing for safety, usability, hygiene, and ease of use. It demonstrated high clinical acceptability among NICU staff and caregivers, who found it easy to incorporate into routine care. This “Postural Supporting Device” is registered with design no. 403807-001 (Figure 1).

**Figure 2:**
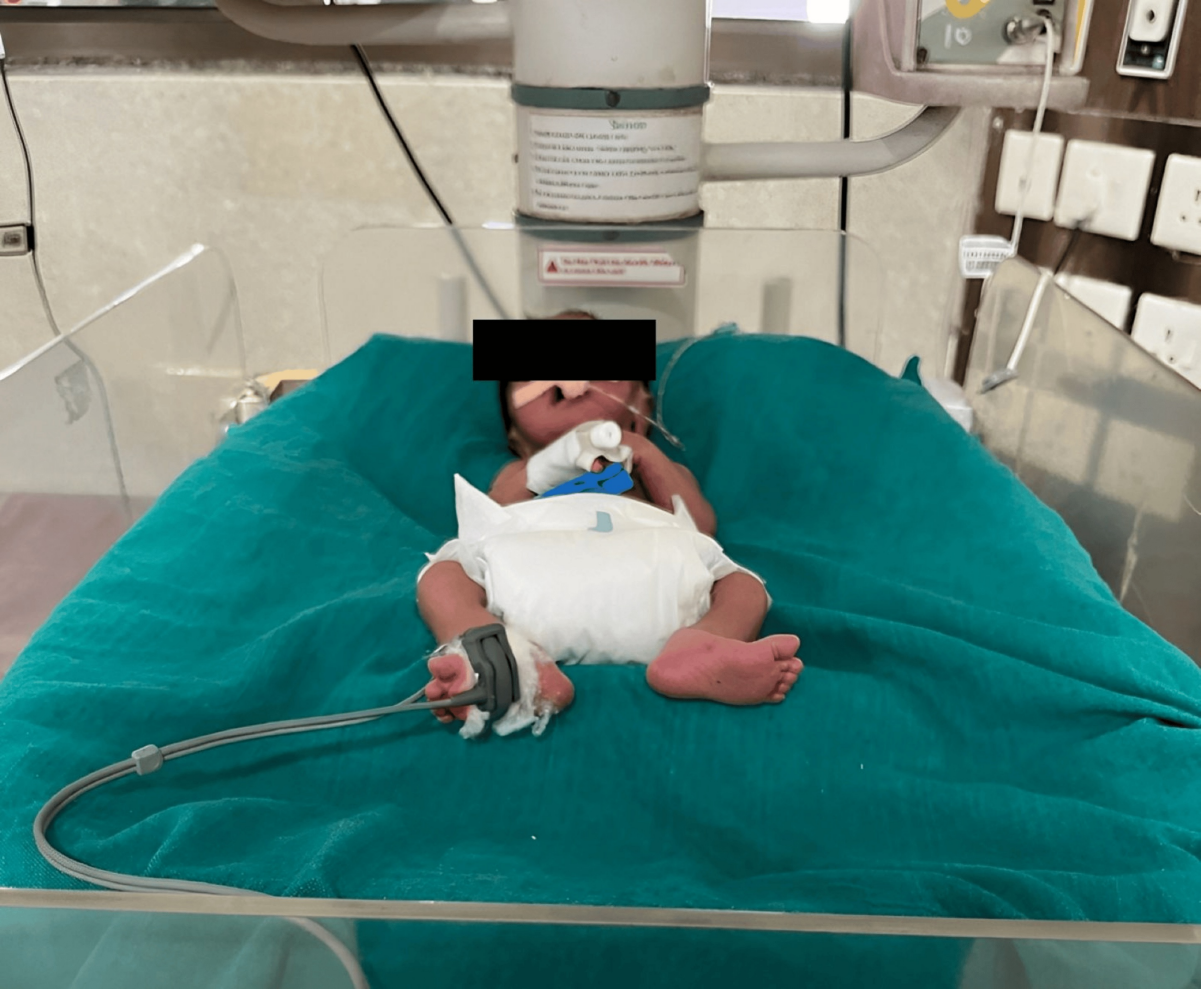
Preterm neonate positioned in the postural supporting device

Figure 2 shows a preterm neonate positioned in the postural supporting device.

The collected data were initially entered into Microsoft Excel (Microsoft Corporation, Redmond, WA, USA). After data cleaning, they were imported into IBM SPSS Statistics for Windows, Version 25.0 (Released 2017; IBM Corp., Armonk, NY, USA) for analysis. Both descriptive and inferential statistical methods were employed. The Wilcoxon test was used for within-group comparisons, with a p-value of less than 0.05 considered statistically significant.

## Results

This study included nine preterm neonates with a mean gestational age of 32.4 ± 1.2 weeks. Of these, five (55.55%) were male and four (44.45%) were female. Only one neonate (11.12%) required oxygen support. The mean birth weight was 1.57 ± 0.28 kg, indicating that all neonates were classified as having low birth weight (Table 1).

**Table 1:** Demographic characteristics of the included preterm neonates

Variable	N (%)/mean ± SD
Male	5 (55.56)
Female	4 (44.45)
Required oxygen	1 (11.12)
Gestational age (weeks)	32.4 ± 1.2
Birth weight (kg)	1.57 ± 0.28

**Figure 3:**
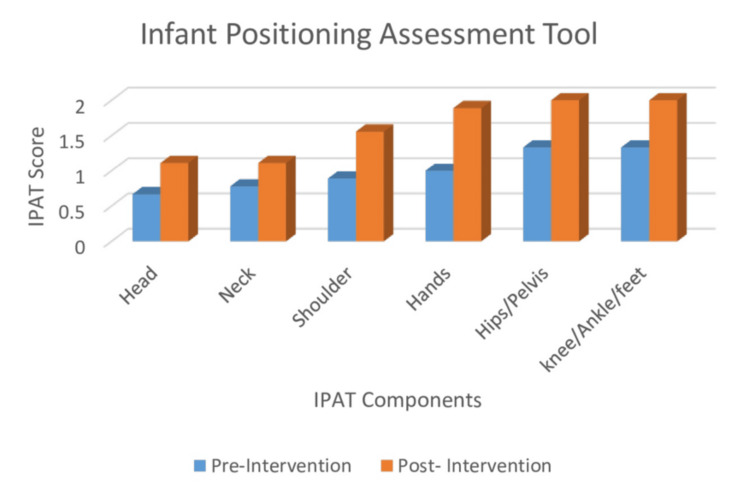
IPAT component score IPAT, Infant Positioning Assessment Tool

Figure 3 presents a component-wise comparison of pre- and post-intervention scores on the IPAT, which evaluates six key body regions. Post-intervention scores were consistently higher across all components, indicating improved postural alignment following the use of the postural support device. Modest improvements were observed in the head and neck regions, while the shoulders and hands showed more pronounced gains. The most substantial improvements were noted in the hip/pelvis and knee/ankle/feet regions.

The results reflect enhanced midline orientation and flexion patterns across all assessed areas. This consistent improvement supports the feasibility and potential benefit of the newly developed postural support system in enhancing the overall quality of neonatal positioning.

A Wilcoxon signed-rank test was conducted to compare pre- and post-intervention scores. The mean pre-intervention score was 6.16 ± 2.99, with a median of 6. Post-intervention, the mean score increased significantly to 10.5 ± 1.22, with a median of 11 (Table 2). The analysis revealed a statistically significant improvement in IPAT scores following the intervention (Z = -2.232, p = 0.026), indicating that the postural support device had a positive impact on the infants’ postural alignment.

**Table 2:** Pre- and post-intervention scores of the IPAT IPAT, Infant Positioning Assessment Tool

Group	Mean ± SD	SEM	Median	p-Value	z-Value
Pre-intervention	6.16 ± 2.99	1.22	6	0.026	-2.232
Post-intervention	10.5 ± 1.22	0.5	11		

## Discussion

This feasibility study aimed to evaluate the effectiveness of a newly designed postural support device in enhancing postural development in NICU-admitted preterm neonates. After one week of continuous use of the device alongside neonatal physiotherapy interventions, a noticeable and statistically significant improvement was observed in the neonates’ posture. The higher post-intervention IPAT scores indicate improved alignment and overall postural development.

The findings are consistent with existing literature emphasizing the importance of physiological flexion in supporting postural development in preterm infants [8,9]. In utero, a flexed, midline posture is naturally supported; however, preterm birth removes this protective environment, leaving infants more susceptible to poor alignment and postural issues [4,6]. In this study, a meaningful improvement in posture was observed following the use of the novel postural support device. The mean IPAT score increased from 6.16 ± 2.99 before the intervention to 10.5 ± 1.22 after, with a statistically significant p-value of 0.026, suggesting that the device contributed positively to improved postural alignment.

A key strength of this study lies in the design of the device, which was created to mimic the intrauterine position. It provides targeted support at the head, shoulders, pelvis, and lower limbs, promoting a midline flexed posture. This helps to counteract the effects of gravity, which can lead to postural asymmetries and extension patterns in preterm infants [15]. The device offers both adjustability and stability, making it adaptable for various sizes and weights of preterm infants. Importantly, the device is practical for NICU use, allowing for routine medical care, temperature monitoring, and feeding while maintaining hygiene and safety standards. Its user-friendly design also enables parents to easily reposition their infants into a flexed posture after each feed. Involving parents in neonatal care has been shown to positively impact both clinical outcomes and parent-infant bonding [16].

While the study demonstrated improved postural outcomes with the new device, it did not include a direct comparison with other available postural support devices or nests. Commercial positioning tools such as nests, rolled blankets, and other aids are commonly used in conjunction with interventions like Kangaroo Mother Care, feeding support, and routine handling. These practices are known to improve infant comfort and positioning. Future research comparing this device with existing supports would be valuable to establish its relative efficacy and clinical utility.

Despite the promising results, this study has limitations. The small sample size (n = 9) limits the generalizability of the findings. Additionally, the absence of a control group makes it difficult to compare the intervention with traditional positioning methods. Moreover, this study focused only on short-term postural outcomes and did not assess long-term developmental impacts, which remain an important area for future investigation.

Nevertheless, the encouraging findings underscore the potential benefits of this approach in promoting postural development in preterm neonates. Especially in settings with limited access to costly commercial products, this simple, cost-effective, and clinically compatible device may offer a practical solution for enhancing neonatal care in the NICU.

## Conclusions

This feasibility study demonstrates that a novel postural support device can significantly improve postural alignment in preterm neonates. Improvements in both component-specific and overall IPAT scores suggest the device is effective, well-tolerated, and suitable for clinical use.

Although the sample size was small, consistent improvements across all body regions indicate that the device may support better neuromuscular and musculoskeletal development in preterm infants. These findings highlight the need for larger-scale studies with long-term follow-up to validate the observed benefits and further investigate the broader developmental impact of such interventions.
